# Fruitlet carbohydrate status and relative growth rate explain apple abscission rates irrespective of flower position

**DOI:** 10.3389/fpls.2026.1876299

**Published:** 2026-06-24

**Authors:** Laura A. Hillmann, Thomas D. Sharkey, Todd C. Einhorn

**Affiliations:** Michigan State University, East Lansing, MI, United States

**Keywords:** crop load, non-structural carbohydrate, sorbitol, source sink characteristics, thinning

## Abstract

The setting of apple (*Malus* x *domestica* Borkh.) fruitlets is primarily determined by their relative sink strength, which depends on the balance between carbohydrate supply and demand and the number of competing sinks. It is generally accepted that limits in carbohydrate supply predispose small fruitlets, i.e., weak sinks, to abscission. This study examined the relationship among carbohydrate status, fruitlet growth, and abscission in ‘Gala’ and ‘Honeycrisp’ over two seasons. Fruitlets were sampled every other day after chemical thinning applications according to their flower position in the cyme (king vs. lateral) and absolute fruitlet weights and compared to untreated controls. At each sample date, fruitlets were selected from three distinct size classes: 90^th^, 50^th^, and 10^th^percentile of the sample population, each with an associated fruit set prediction according to the fruitlet size distribution model (FSDM). Soluble sugars (glucose, fructose, sucrose, sorbitol) and starch were quantified at each sample date. Small fruitlets (10^th^ percentile) consistently exhibited reduced soluble sugar concentrations and relative growth rates prior to abscission, while larger fruitlets (50^th^and 90^th^ percentiles) maintained higher sugar levels; thus, having greater sink strength and a higher retention probability. Sorbitol and sucrose concentrations were often dynamic and generally reflected modeled tree-level carbon balance, based on the MaluSim model; these sugars also declined markedly after chemical thinner applications were made indicating stress. Starch accumulation further distinguished stronger from weaker sinks, with exponential accumulation in 90^th^percentile fruitlets under thinning stress. Positional effects were evident, as king fruitlets generally maintained higher sugar concentrations than laterals; however, this effect was confounded by king fruit generally being larger. When position was ignored, fruit weight could explain a fruitlet’s abscission fate. When the FSDM predictions were within 90% of the actual fruit set, fruitlets destined to abscise had markedly reduced concentrations of soluble sugars and lower growth rates than those predicted to be retained, before any visible signs of fruit abscission were apparent. These data underscore the physiological basis of abscission by highlighting the central role of carbohydrate availability and sink strength that determine fruitlet survival, supported by the FSD model

## Introduction

Fruit development in apple (*Malus* x *domestica* Borkh.) is driven by coordinated genetic, hormonal, and environmental regulation, with carbohydrate availability playing a central role in determining fruit growth dynamics and retention (Lakso and Johnson, 1990; Bangerth, 2000). During early season growth, until ~3 to 4 weeks after bloom, cell division disproportionately contributes to fruit growth and the number of cells in the cortex is highly associated with final fruit size (Denne, 1960; Bollard, 1970; Lakso and Goffinet, 2017). Concomitantly, respiration rate of fruitlets is high, reflecting the high metabolic demand of rapid growth (Lakso and Johnson, 1990; Berüter and Droz, 1991). Growth limitations due to interruptions in carbohydrate supply during this exponential growth phase are irreversible, as fruit with fewer cell numbers cannot compensate for their reduced size potential through cell expansion (Goffinet et al., 1995; Lakso et al., 2001). Under favorable conditions, fruitlets maintain a steady growth rate until harvest, but environmental conditions and resource limitations can cause temporary growth cessation ultimately leading to fruitlet abscission (Byers et al., 1991; Lakso et al., 2001).

Generally, only ~5% of apple fruitlets are retained ~ one month from bloom due, in part, to competition among multiple organs (developing leaves, shoots, spurs, and fruitlets) for a finite supply of assimilates (Quinlan and Preston, 1971; Bepete and Lakso, 1998; Reyes et al., 2016). Developing shoot tips are dominant sinks early in the season, and until leaves reach ~50% of full expansion, carbohydrate supply to the fruitlets comes from leaves of spurs and short shoots (Hansen, 1971; Corelli-Grappadelli et al., 1994; Lakso and Goffinet, 2017). The capacity to import sugars depends on the combined effect of metabolic and osmotic sinks. Metabolic activity will lead to higher import of sugars, which in turn leads to a greater osmotic draw to sustain rapid growth (Berüter and Droz, 1991; Celton et al., 2014; Loescher and Everard, 2017). By one month after bloom, larger fruitlets are ~20–25 mm in diameter and become more tolerant to resource supply limitations given the accrual of starch reserves and developed seeds and thus resist chemical thinning (Hansen, 1977; Greene, 2002; Reyes et al., 2016). Thus, the application of crop load management practices during early season fruitlet development and growth is highly important to control fruit set and optimize fruit size.

Carbon gain is tightly linked to light interception and leaf area per fruit, both of which change seasonally as canopy development progresses. Crop load management strategies such as artificial spur extinction (ASE) or chemical thinning reduce within-tree competition by adjusting spur density and flower/fruitlet number and improve canopy light interception (Breen et al., 2016; Penzel et al., 2020). These practices can optimize the balance between leaf area and fruit number and enhance the tree’s overall carbon balance to support optimal fruit set (Lakso et al., 2006; Pallas et al., 2016; Lordan et al., 2020). Similarly, competition between fruitlets of the same cluster can affect fruit set and abscission. The central ‘king’ fruitlet often has higher set rates and faster growth rates than laterals, though thinning response may depend more on size than position (Marini, 2003; Serra et al., 2022). The advantages of the central ‘king’ fruitlets over lateral fruitlets have been linked to improved auxin transport through apical dominance (Bangerth et al., 2000), developmental time (i.e. early flowering and seed set) (van Doorn and Stead, 1997; Yoder et al., 2013), and additional amphivasal vascular bundles within the pedicel (Celton et al., 2014). When competition over resources is reduced, the early growth advantages of king fruitlets are less pronounced (Ferree et al., 2001). Fruit set prediction models like the fruit growth rate (FGR) and the fruitlet size distribution (FSD) models exploit this relationship to predict fruit set by comparing relative growth or size among fruitlets prior to visible abscission (Greene et al., 2013; Hillmann et al., 2025).

In apple, sorbitol and sucrose are the primary phloem-translocated carbohydrates (Loescher, 1987; Moing et al., 1992). Sorbitol is synthesized in source leaves and catabolized in sink tissues by sorbitol dehydrogenase, while sucrose is hydrolyzed by invertases or cleaved by sucrose synthase depending on tissue demand (Loescher and Everard, 2017). These sugars support rapid fruit growth and accumulate in vacuoles as hexoses (Yamaki, 1984). The metabolism and partitioning of translocated sugars are influenced by developmental stage, environmental stress, and sink activity, and plays a central role in determining fruitlet retention (Berüter et al., 1997; Cheng et al., 2005; Li et al., 2018).

During early fruit development, carbohydrate availability is closely linked to growth and retention, with fruitlet competitiveness determined by sink strength—the product of sink size and sink activity. Sink activity reflects both metabolic processes, such as the enzymatic conversion of sorbitol and sucrose to hexoses and starch, and osmotic processes, from sugar accumulation in vacuoles that drive turgor and cell expansion (Loescher and Everard, 2000, 2017). Fructose is the predominant vacuolar sugar in apple fruit and a major contributor to growth because of its strong osmotic effect and sustained accumulation during development (Berüter, 2004; Li et al., 2018; Tijero et al., 2021). The balance between metabolic and osmotic sinks therefore underpins fruitlet sink strength and shapes competition for assimilates, ultimately influencing fruit set outcomes. Signals of nutritional shortage and sugar starvation can lead to an accumulation of sucrose, previously shown to be an adaptive response to stress (Roitsch, 1999; Botton et al., 2011). In this regard, work on developing seeds has shown that high production of auxin causes significant increases of invertase, leading to increased hydrolysis of sucrose to free glucose and fructose which in turn promotes continued import of sucrose, and results in a stronger sink (Yamaki and Asakura, 1988).

The complex interplay of competition between sink tissues and the assimilation of carbohydrates has a pronounced effect on fruit set and abscission processes. Chemical thinner applications with synthetic auxins, such as naphthalene acetic acid (NAA) are proposed to have several modes of action, including stimulation of ethylene production (Curry, 1991b; Zhu et al., 2008; 2010), inhibition of photosynthesis (Stopar et al., 1997; Zhu et al., 2011), disruption of auxin transport (Ebert and Bangerth, 1982), and reduced carbohydrate transport to fruitlets (Schneider, 1975). Thus, the balance of carbohydrate supply and demand plays an important role in determining fruit abscission and thinning efficacy (Lordan et al., 2020). With many factors influencing fruit set and abscission, growers rely on prediction models to guide crop load management practices. The MaluSim model is used to estimate whole tree carbon balance and provides insights into the timing, dose, and type of thinning compound to apply (Lakso, 2011). Additionally, fruit set prediction models such as the fruit growth rate (FGR) or the fruitlet size distribution (FSD) model have been developed to help growers assess thinner efficacy and can help growers decide if additional applications are necessary to achieve a target fruit set (Greene et al., 2013; Hillmann et al., 2025).

While these models are valuable decision-support tools, they describe and predict fruitlet abscission but do not assess the physiological mechanisms linking carbohydrate availability, sink strength, and fruit set. In this study, we aimed to connect fruit set predictions and fruitlet growth to the carbohydrate status of individual fruitlets of ‘Gala’ and ‘Honeycrisp’. We characterize the relationship between fruitlet traits such as position, size and carbohydrate availability, ultimately to inform emerging fruit set prediction tools and refine thinning strategies.

## Materials and methods

### Plant material

Experiments conducted in 2021 and 2022 utilized apples from well-established, uniform, tall spindle orchards planted at a density of 2941 trees per hectare (1 m x 3.4 m spacing). Orchards were located at the Michigan State University Clarksville Research Center in Clarksville, Michigan (lat. 42.9° N, long. 85.3° W) on a sandy-clay loam soil: 6th leaf ‘Gala Baigent’ (Brookfield™) on ‘Budagovsky 9’ (Bud 9) (2021), 5th leaf ‘Gala Simmons’ (Buckeye™) on ‘Bud 9’ (2022) and 6th leaf ‘Honeycrisp BAP 2000’ (Firestorm™) on ‘Geneva 11’ (CG11) (2022). Except for thinning treatments, all horticultural inputs (nutrition, irrigation, pruning and training) and pest management practices were conducted to maintain plants in a non-limited, stress-free condition.

### Treatments and experimental design

In 2021, seventy healthy trees were selected based on trunk circumference (12 cm +/- 10%) measured 20 cm above the graft union and uniformity (canopy volume and bloom) and divided between two treatments in a complete randomized design (CRD) arrangement: a non-thinned control and a chemically thinned treatment using 15 mg· L^-1^ NAA applied at ~ 6 mm fruitlet diameter. There were five replicates comprising seven contiguous trees per replicate. In 2022, only chemically thinned trees were evaluated (35 trees per cultivar with 5 replicates of seven contiguous trees). Chemical thinning varied between cultivars: 10 mg· L^-1^ NAA + 565 mg· L^-1^ Carbaryl or 5 mg· L^-1^ NAA + 565 mg· L^-1^ Carbaryl for ‘Gala’ and ‘Honeycrisp’, respectively. Thinners were applied at 6 mm fruitlet diameter and again at 12 mm fruitlet diameter at the same concentrations. At bloom, separate sets of spurs were tagged on well exposed limbs to either generate fruit set predictions or be sampled for carbohydrate analysis (further described below).

### Fruit set and MaluSim predictions

Fruit set predictions were generated using the fruitlet size distribution (FSD) model (Hillmann et al., 2025; Hillmann and Einhorn, 2024) using the same trees as described above. Twenty spurs per tree were tagged for sampling which was conducted every two days after thinner application (n=100 spurs per sample date). Each tree was only sampled once to avoid potential confounding effects of cumulative spur extinction (sampling) on fruit set. Thus, following each of the seven sampling dates, the sampled tree was excluded from the remainder of the experiment. All individual fruitlets on the spurs were weighed and automatically sorted by mass using an Excel sheet. The FSD model generated a fruit set prediction for each sampling day, thus creating a series of prediction points. For each treatment, final fruit set was recorded at harvest from 20 spurs per replicate tree (n=100), preselected at bloom. Fitted curve functions were used to generate the exact day final fruit set was predicted by the model (Hillmann et al., 2025).

Green tip date and full bloom date were entered into the Network for Environmental and Weather Applications (NEWA) website containing the MaluSim application (www.malusim.org) to generate predictions on the carbohydrate balance of trees as previously described by Lakso (1997).

### Sampling for carbohydrate analysis

In 2021, fruitlets were sampled every two days for a period of ~15 days, commencing on the day of thinner application. On each sampling date, twenty pre-selected, tagged spurs were collected from one tree per rep and treatment for carbohydrate analysis, immediately placed on dry ice, transported to the lab and stored at -80 °C. Fruitlets from spurs were weighed and sorted by weight (g) according to the Fruitlet Size Distribution (FSD) model (Hillmann et al., 2025). Five fruitlets from each of the 5 replicates representing each of three size classes (10^th^, 50^th^, and 90^th^ percentile of entire sample population for the given date according to mass) were selected. Thus, a total of 25 fruitlets per treatment and size class were represented on each sample date.

In 2022, sampling was again conducted alongside the sampling for the FSD model predictions. A step was added to immerse fruitlets in liquid nitrogen immediately following weighing to limit metabolism. Fruitlets were then stored at -80 °C until processing. Additionally, fruitlets were sorted separately based on their position in the cymose inflorescence as either ‘king’ or lateral fruitlets. Thus, in 2022, a total of 25 fruitlets per cultivar, fruitlet position, and size class were considered.

### Carbohydrate analysis

Samples were ground to a fine powder by hand under sub-freezing temperatures using a mortar and pestle. The powder was placed into 2 ml Eppendorf tubes and weighed on a precision balance (XSR105 DualRange, Mettler Toledo LLC, Columbus, OH, USA). Soluble sugars (glucose, fructose, sucrose) and starch were extracted using a perchloric acid extraction method (Loreto and Sharkey, 1993). Starch was analyzed by enzymatic digestion to glucose using α-amylase (cat. no. 700004188, Megazyme International Ireland, Bray, Ireland) and α-amyloglucosidase (cat. no. 9001427, Megazyme International Ireland, Bray, Ireland).

Soluble sugars (glucose, fructose, and sucrose) were quantified enzymatically using a coupled assay in 96-well microplates (adapted from Lowry and Passonneau, 1972) with a spectrophotometer (Multi-mode Microplate Reader, FilterMax F5, Molecular Devices, LLC., San Jose, CA, USA).

A 150 mM HEPES buffer solution at pH 7.2 was prepared. To minimize pipetting error and improve consistency, a reaction premix was prepared in a 15 -mL centrifuge tube with the following components (scaled to the number of wells, n): 190 μL × n HEPES buffer (final 110 mM in well), 5 μL × n nicotinamide adenine dinucleotide phosphate (NADP^+^; final 1.282 mM per well), 5 μL × n adenosine triphosphate (ATP; final 250 nmol per well) and 5 μL × n Glucose-6-phosphate dehydrogenase (G6PDH, cat. no. G8529, final 0.4 Units per well). Samples (5 μL) were pipetted into individual wells (performed in triplicates). Subsequently, 200 μL of the reaction premix was added to each well using a multichannel pipette, taking care not to disturb the sample. The plate was placed into the microplate reader, and a kinetic assay initiated at 340 nm. The plate reader’s mixing function was used to homogenize well contents prior to measurement. Absorbance was recorded continuously until a stable baseline was obtained. This measured any glucose 6-phosphate in the sample and allowed the readings to stabilize.

To measure glucose, hexokinase (HXK, cat. no. H4502, 5 μL per well, final 1 Unit) was loaded on to a multichannel pronged tool, allowing simultaneous addition of the enzyme to all samples once the baseline stabilized. The following reaction was monitored until a plateau was reached, and both the baseline and plateau phases were averaged to calculate net absorbance change. The extinction coefficient used for NADPH was 6220 L mol^-1^ cm^-1^.

For determination of fructose and sucrose, first 2 μL and 10 μL of phosphoglucose isomerase (PGI, cat. no. P9544, final 2 Units) was added and then invertase (INV, cat. no. I4504, 50 U) (Sigma-Aldrich, St. Louis, MO, USA), in the same manner described above. For sucrose quantification, the microplate was incubated at 37 °C in the spectrophotometer after addition of invertase.

Sorbitol was measured separately using the same equipment with an assay kit (cat. no. K-SORB) purchased from Neogen^®^ (MEGAZYME International Ireland, Bray, Ireland). The increase of absorbance of INT-formazan was read at 492 nm, proportional to the concentration of sorbitol.

### Statistical analysis

Data were analyzed using a three-way analysis of variance (ANOVA) with treatment, time point, and percentile as fixed factors. Prior to analysis, model assumptions were assessed by examining residual plots and testing normality using the Shapiro-Wilk test. Because fruitlets were destructively sampled, no individual fruitlet was measured more than once.

When significant main effects or interactions were detected, *post-hoc* analyses were conducted using estimated marginal means (EMMs). Pairwise comparisons were performed among treatment levels within each time point × percentile-class combination, among time points within each treatment × percentile-class combination, and among percentile classes within each treatment × time-point combination, as appropriate. Tukey’s HSD adjustment was applied to control the family-wise error rate associated with multiple comparisons.

All analyses were performed using R statistics program (version 4.4.0 (2024-04–24 ucrt) “Puppy cup”) using the emmeans package for *post-hoc* comparisons. Statistical significance was set at α = 0.05. Figures display observed means ± standard error.

## Results

**Figure 1:**
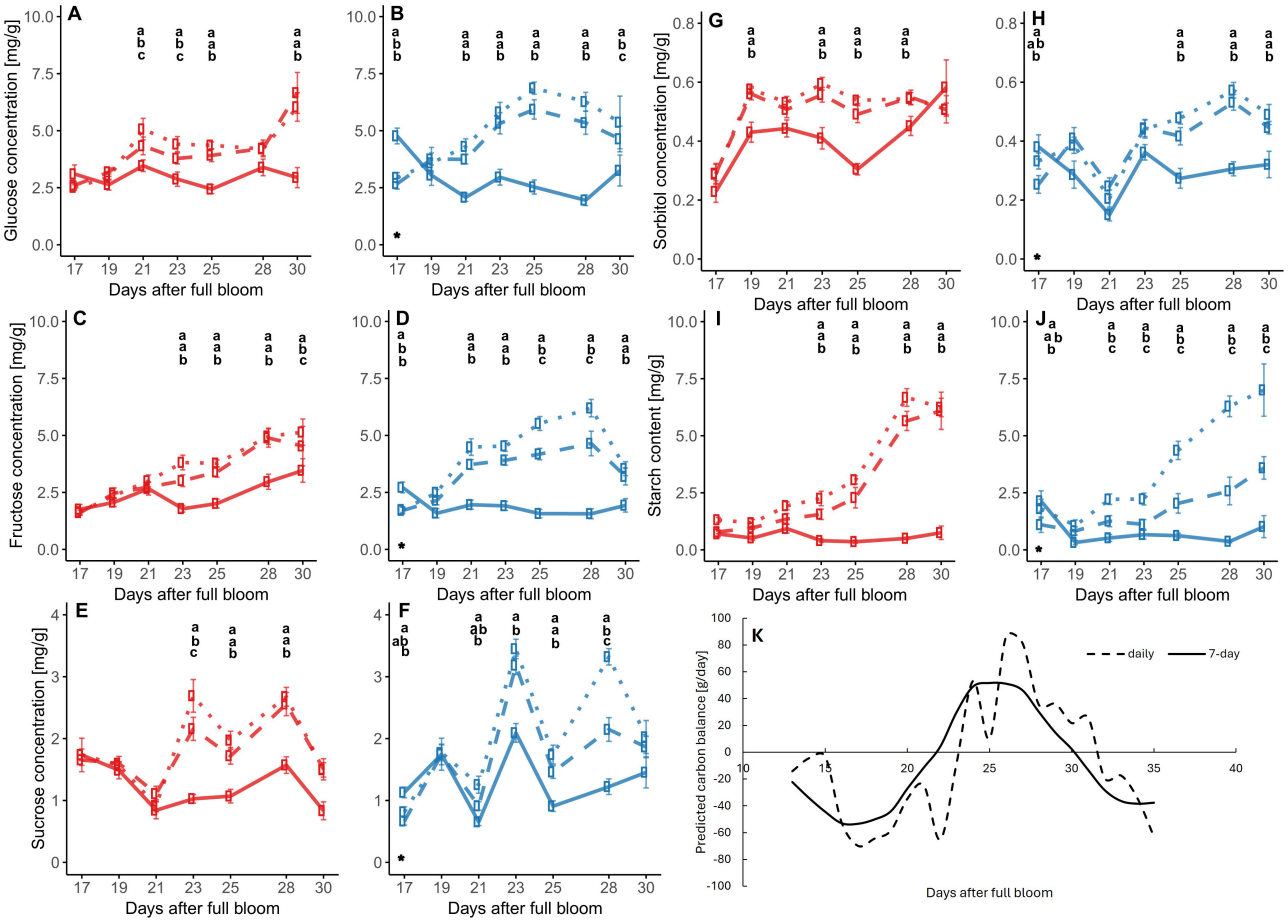
Soluble sugar concentrations **(A–H)** and starch content **(I, J)** of ‘Gala’ apple fruitlets in 2021 and predicted carbon balance by MaluSim model **(K)**. Sugars are shown for Unthinned control (red panels) and thinned treatment (blue panels). Solid lines represent the 10^th^ percentile, dashed lines the 50^th^ percentile, and dotted lines the 90^th^ percentile of the fruitlet population. Asterisks indicate day of thinner application in the thinned treatment. Error bars represent the standard error around the mean (n=25), different letters indicate significant differences between means on the same sample date based on Tukey’s HSD.

Significant effects of treatment, time, and fruitlet size on soluble carbohydrate and starch concentrations were detected (p<0.001). In 2021, glucose was the predominant soluble sugar, reaching 2–8 mg g^−1^ FW in both thinned and unthinned treatments (**Figures 1A, B**). Fruitlets in the 50^th^ and 90^th^ percentiles consistently had higher glucose concentrations than those in the 10^th^ percentile, with differences becoming pronounced at 21 d after full bloom (DAFB) (or 5 d after thinner in the thinned treatment) and persisting until the end of the sampling period. In the control, the same pattern was evident, except at 28 DAFB when concentrations of sorbitol and glucose converged across all size classes (**Figures 1B, H**). Fructose followed a similar pattern, albeit at slightly lower concentrations. In the thinned treatment, fructose concentrations of 90^th^ percentile fruitlets dropped sharply at 30 DAFB, while 10th percentile fruitlets remained stable; in contrast, concentrations in the control increased steadily across all size classes (**Figures 1C, D**).

Sucrose accumulated at lower levels overall and showed greater fluctuation, often mirroring carbon balance estimates from the MaluSim model (**Figures 1 E, F K**). Across both treatments, 10^th^ percentile fruitlets generally had the lowest sucrose concentrations (**Figures 1 E, F**). Sorbitol was the least abundant sugar (0.1–0.6 mg g^−1^ FW) but exhibited fluctuations similar to glucose. In response to chemical thinning, sorbitol levels dropped sharply across all size classes at 21 DAFB (6 days after thinner, DAT) but recovered quickly, except in the 10^th^ percentile where concentrations remained significantly lower. The FSD model had generated a fruit set prediction 6 DAT for thinned trees. Conversely, sorbitol concentration had no appreciable change in the unthinned control, although 10^th^ percentile fruitlets had markedly lower concentrations on 25 DAFB concurrent with a predicted deficit in the estimated carbon balance. Predicted fruit set of unthinned control trees required two additional days compared to the thinned treatment; concomitantly, soluble sugar concentrations of 10^th^ percentile fruitlets fell below those of 90^th^ and 50^th^ percentile size classes on 23 DAFB. Starch remained consistently low in 10^th^ percentile fruitlets across treatments (**Figures 1 I, J**). Larger fruitlets, however, accumulated starch significantly from 23 DAFB onward, with exponential accumulation in 90^th^ percentile fruitlets of the thinned treatment, while 50^th^ percentile fruitlets increased more moderately. Notably, starch accumulation between fruitlets in the 90^th^ and 50^th^ percentiles did not show differences from the unthinned control (**Figure 1J**).

**Figure 2:**
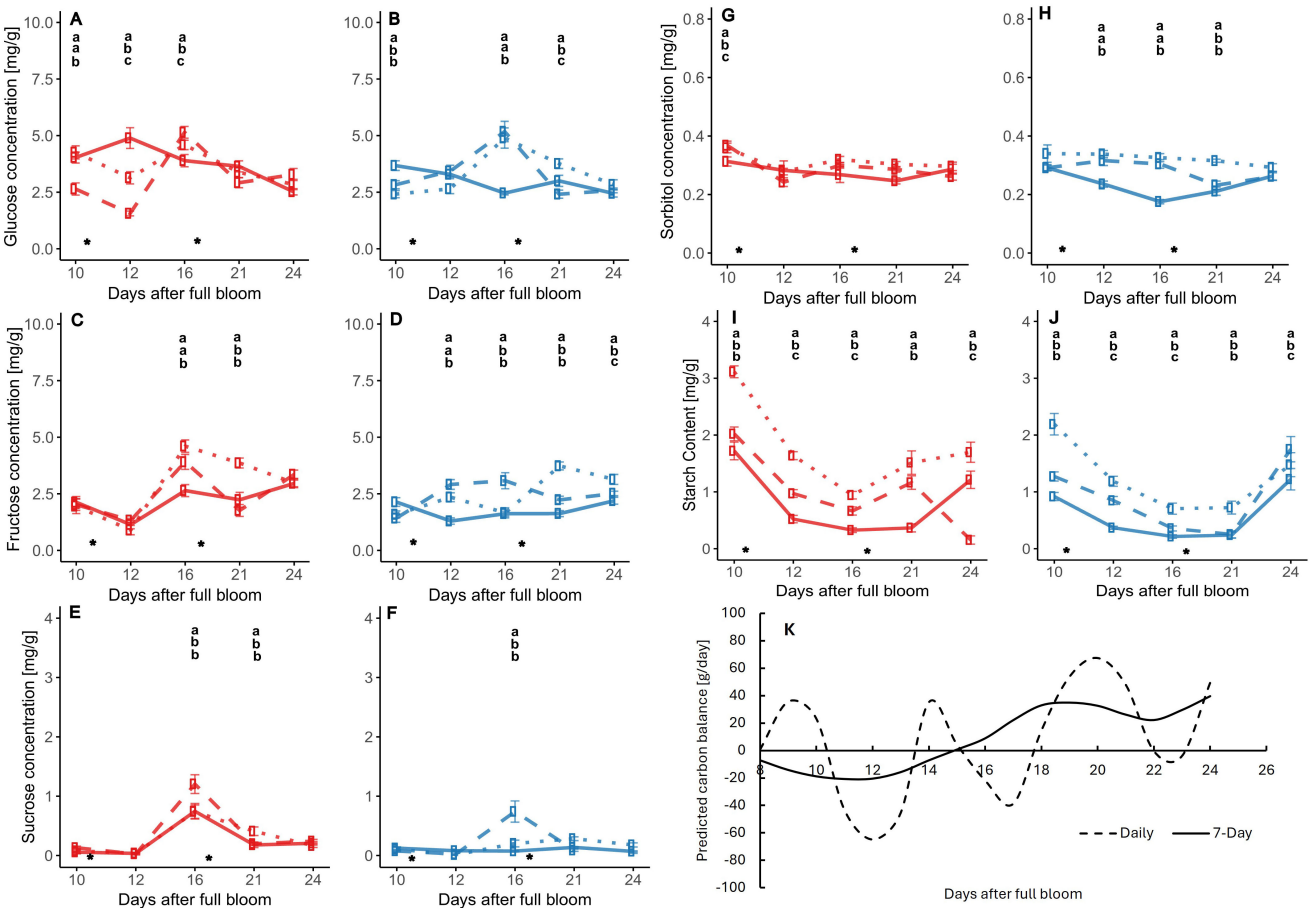
Soluble sugar concentrations **(A–H)** and starch content **(I, J)** of ‘Gala’ apple fruitlets in 2022 and predicted carbon balance by MaluSim model **(K)**. Sugars are shown for king fruitlets (red panels) and lateral fruitlets (blue panels). Solid lines represent the 10^th^ percentile, dashed lines the 50^th^ percentile, and dotted lines the 90^th^ percentile of the entire population. Asterisks indicate day of thinner applications. Error bars represent the standard error around the mean (n=25), different letters indicate significant differences between means on the same sample date based on Tukey’s HSD.

**Figure 3:**
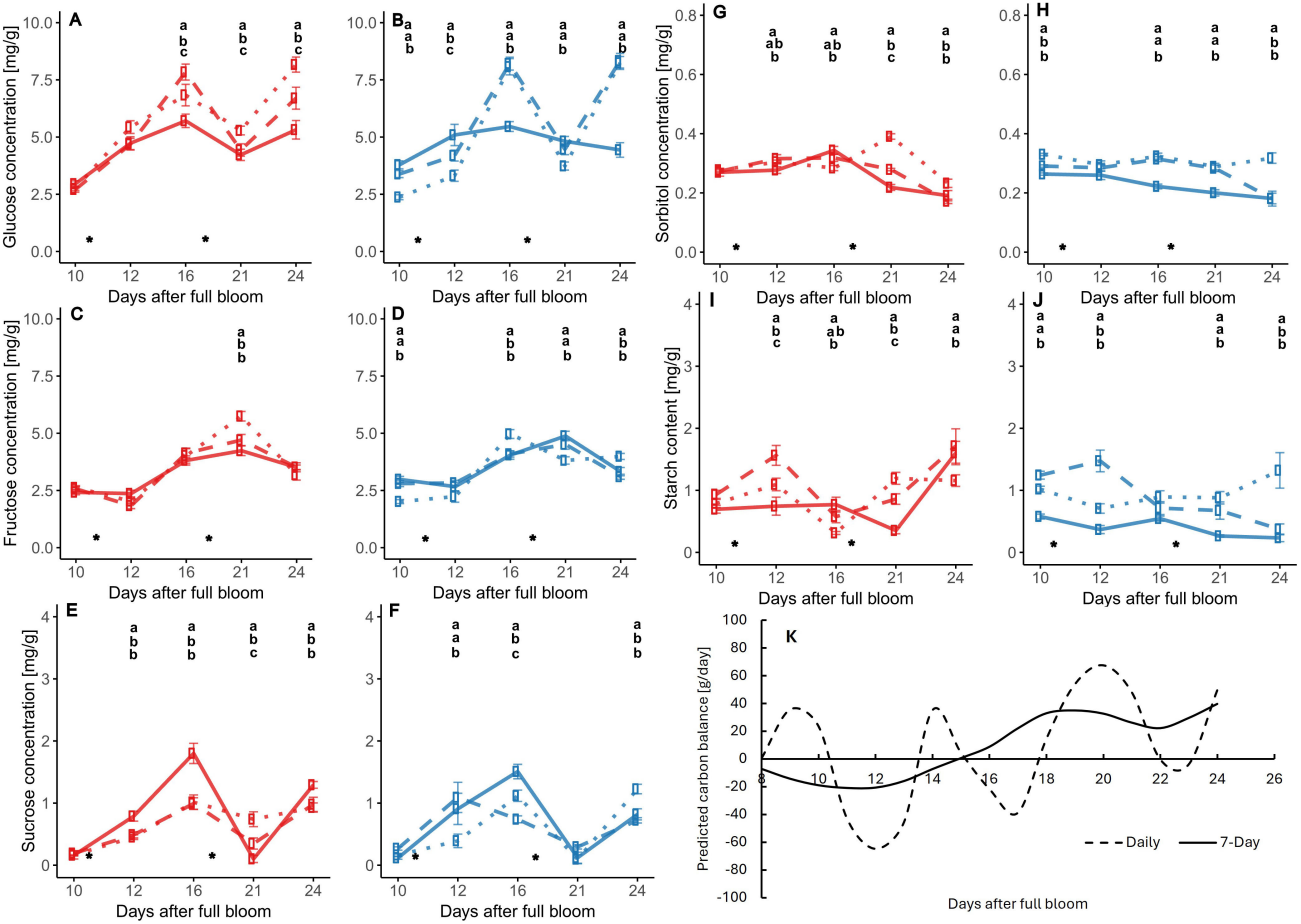
Soluble sugar concentrations **(A–H)** and starch content **(I, J)** of ‘Honeycrisp’ apple fruitlets in 2022 and predicted carbon balance by MaluSim model **(K)**. Sugars are shown for king fruitlets (red panels) and lateral fruitlets (blue panels). Solid lines represent the 10^th^ percentile, dashed lines the 50^th^ percentile, and dotted lines the 90^th^ percentile of the entire population. Asterisks indicate day of thinner applications. Error bars represent the standard error around the mean (n=25), different letters indicate significant differences between means on the same sample date based on Tukey’s HSD.

In 2022, soluble sugars and starch were generally lower in both cultivars than in 2021 (**Figures 2**, **3**). In king fruitlets of ‘Gala’, glucose concentrations were initially higher in 10^th^ percentile fruitlets, but differences disappeared following the second thinner application at 16 DAFB (**Figure 2A**). Fructose showed the opposite trend, with 90^th^ and 50^th^ percentile fruitlets accumulating at higher rates early in development, as was similarly observed in 2021 (**Figures 1**, **2**). A spike in sucrose across all size classes and both cultivars was observed at 16 DAFB, coinciding with a modeled increase in the estimated daily carbon balance (**Figures 2**, **3K**). Sorbitol concentrations did not differ with respect to fruitlet size in the king fruitlet population, however, the 10^th^ percentile of lateral fruitlets had consistently lower concentrations than 50^th^ and 90^th^ percentile classes, for both cultivars (**Figures 2**, **3**). By 21 DAFB (shortly after the second thinner application), the FSD model predicted final fruit set in both cultivars coinciding with a decline in sorbitol in 10^th^ percentile king and lateral fruitlets. Notably, a portion of the 10^th^ percentile fruitlets of ‘Gala’ regained sorbitol concentrations to former levels at 24 DAFB (**Figures 2G, H**). Starch accumulation was consistently greatest in 90^th^ percentile fruitlets across both positions, though the pattern was not exponential as in 2021 (**Figures 1**, **2**). Interestingly, differences in starch content of ‘Honeycrisp’ among fruitlet positions were more distinct than ‘Gala’, whereby 10^th^ percentile lateral fruitlets accumulated less starch compared to king fruitlets (**Figures 3I, J**).

**Figure 4:**
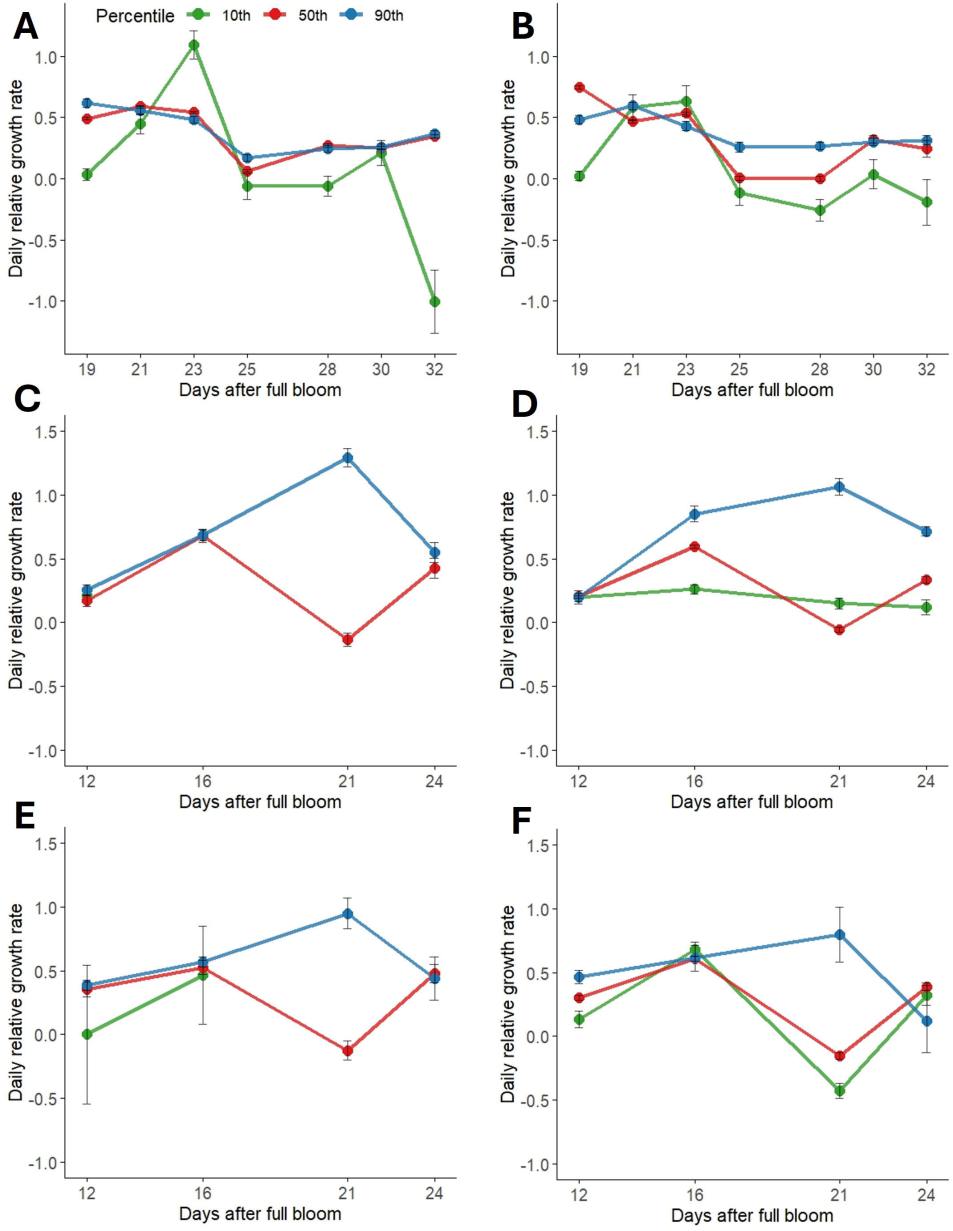
Daily relative growth rates of ‘Gala’ fruitlets in the unthinned control **(A)** and thinned treatment **(B)** in 2021. In 2022, relative growth rates are shown for ‘Gala’ king **(C)** and lateral **(D)** fruitlets, and for ‘Honeycrisp’ king **(E)** and lateral **(F)** fruitlets. Green lines indicate the 10^th^ percentile fruitlets, red lines the 50^th^ percentile, and blue lines the 90^th^ percentile.

**Figure 5:**
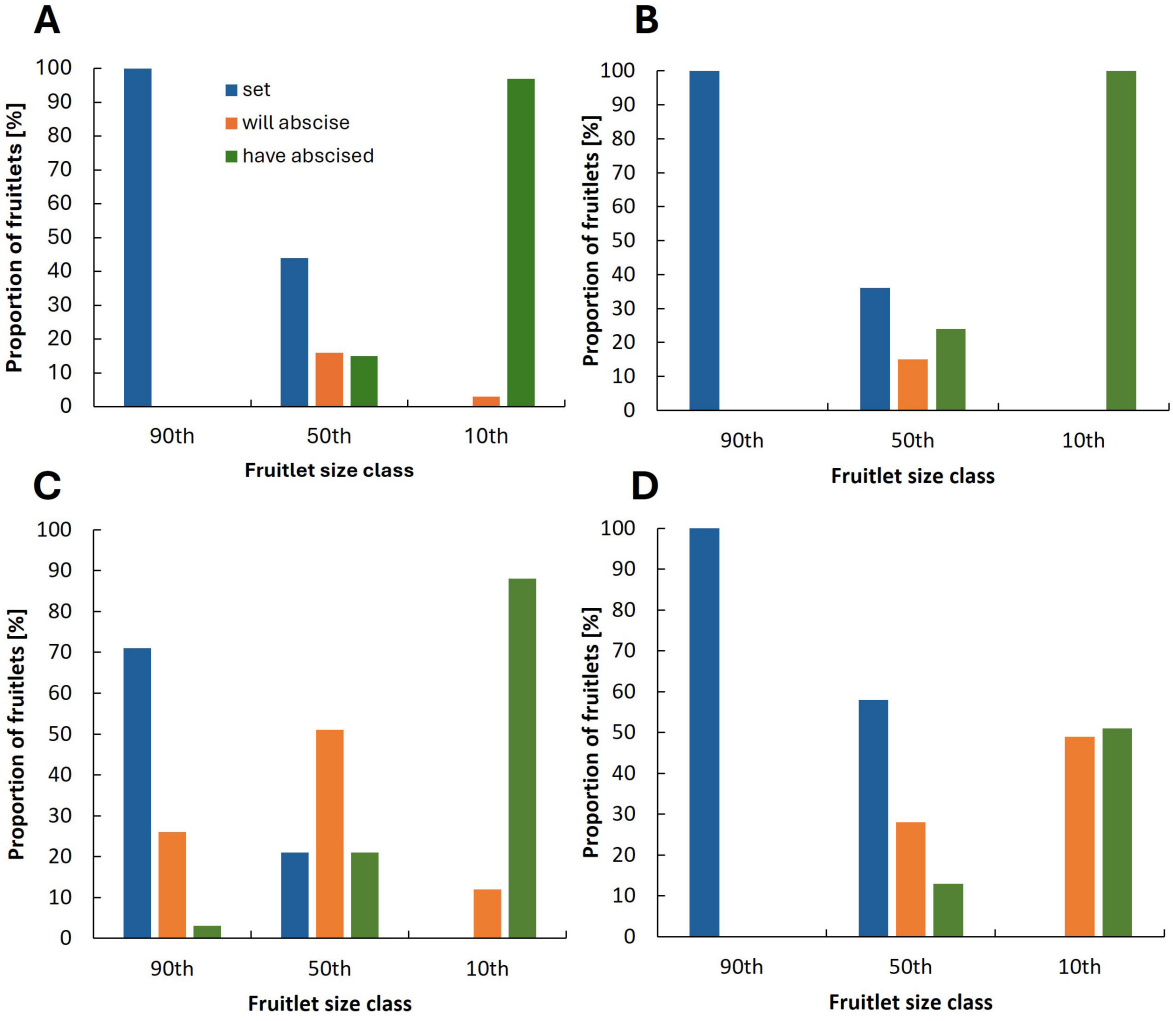
Proportion of fruitlets that set or abscise at the time of prediction by the FSD model. The two upper panels show unthinned **(A)** and thinned **(B)** trees of ‘Gala’ in 2021. The lower panels indicate proportion of ‘Gala’ **(C)** and ‘Honeycrisp’ **(D)** fruitlets in 2022. Fruitlets were grouped based on size percentiles used in the FSD model predictions.

The daily relative growth rate was strongly associated with soluble sugar dynamics. In 2021, 10^th^ percentile fruitlets of the thinned treatment initially grew rapidly between 19 and 23 DAFB, while sorbitol, fructose, and glucose concentrations steadily declined (**Figure 4B**). After 23 DAFB, growth velocity reduced sharply, and both growth and sugar concentrations remained lower in 10^th^ percentile fruitlets. In contrast, 50^th^ and 90^th^ percentile fruitlets maintained steadier growth rates. At 21 and 25 DAFB, reductions in sorbitol and sucrose concentration, respectively, coincided with reduced growth rates. The FSD model predicted final fruit set at 21 DAFB. This was followed by recovery in either growth rate (50^th^ percentile) or stable growth (90^th^ percentile) synchronous with increased sugar accumulation. A similar relationship was observed in the untreated control, although the 50^th^ and 90^th^ percentile fruitlets displayed nearly identical growth rates and sugar accumulation patterns compared to the thinned treatment (**Figures 4A, B**). These relationships were also underscored by abscission and setting rates in the orchard (**Figures 5A, B**). In the unthinned control, fruitlets of the 50^th^ and 90^th^ percentile – exhibiting very similar growth rates- likely contributed to the higher proportion of setting fruitlets compared to the thinned treatment. This difference was especially clear in the proportion of setting fruitlets within the 50^th^ percentile size class of the unthinned control, which was nearly double that of the thinned treatment at the time of FSD model prediction (**Figures 5A, B**).

In 2022, growth patterns were consistent across cultivars and fruitlet positions, with 10^th^ and 50^th^ percentile fruitlets showing a pronounced reduction in growth rate at 21 DAFB, coinciding with reductions in soluble sugars and higher abscission rates (**Figures 4C–F**, **5C, D**). Conversely, fruitlets in the 90^th^ percentile exhibited a peak in growth rate over this period. Notably, king fruitlets in the 10^th^ percentile size class abscised by 16 DAFB (**Figure 5E**). In fact, the majority of the abscising population (50^th^ and 10^th^ percentiles) throughout the sampling period was composed of laterals (data not shown). Soluble sugar concentrations increased in 50^th^ percentile fruitlets when growth was rapid and decreased in synchrony with retarded growth. In the 90^th^ percentile, growth rates remained rapid until 21 DAFB while sugar concentrations first increased and then declined, preempting growth reductions (**Figures 4C–F**).

The FSD model generates fruit set predictions on each sampling date as fruitlets grow and population dynamics shift. With continued sampling, a progressive decline in predicted fruit set is observed until the curve stabilizes and final fruit set is predicted, although additional abscission may still occur. Thus, at the time of the final fruit set prediction, some fruitlets remaining on the tree are already predicted to abscise, while some predicted to persist may still drop later. To evaluate prediction accuracy, the proportion of fruitlets predicted to persist or abscise was compared with absolute fruit set at harvest.

In both cultivars, fruitlets in the 90^th^ percentile indeed set as predicted by the FSD model and persisted through harvest. Conversely, 10^th^ percentile fruitlets had abscised or were predicted to do so (**Figures 5C, D**). In ‘Gala’, at the time of prediction, 51% of 50^th^ percentile fruitlets were predicted to abscise, and persisting fruitlets within this group were accurately classified by the model. In ‘Honeycrisp’, 50^th^ percentile fruitlets exhibited greater size variability and higher predicted fruit set rates (i.e., 58%), but only 6% persisted to harvest (data not shown). Similarly, within the 90^th^ percentile, 57% of the fruitlets predicted to persist abscised and did not persist through harvest. In both cultivars, king fruitlets dominated the 90^th^ percentile population, whereas lateral fruitlets comprised most of the abscising groups (i.e., 50^th^ and 10^th^ percentiles, data not shown).

## Discussion

The objective of this work was to determine if the concentrations of specific sugars correlated with a fruit’s mass and probability to abscise according to a fruit set prediction model (Hillmann et al., 2025). The carbohydrate status of fruitlets during the early post-bloom period is a central determinant of sink strength and retention potential (Berüter and Droz, 1991; Celton et al., 2014). Our results demonstrate that the dynamics of soluble sugar concentrations and starch content are closely aligned with early fruitlet growth and abscission in apple. Across cultivars and years, fruitlets in the 10^th^ percentile consistently exhibited lower sugar concentrations and reduced growth rates prior to abscission, whereas fruitlets in the 90^th^ percentiles maintained higher sugar concentrations and stronger growth rates. Fruitlets in the 50^th^ percentile were often similar to 90^th^ percentile fruitlets while at other times more closely behaving like 10^th^ percentile fruitlets. An estimation of a fruitlet’s abscission fate throughout early development via a predictive model facilitated a practically real-time accounting of a fruitlet’s carbohydrate status with its fruit set fate, before visible signs of abscission were perceptible.

The decline in soluble sugar concentrations in abscising (10^th^ percentile) fruitlets coincided with low relative growth rates in both cultivars and years, suggesting that sugar depletion may act as a limiting factor for sustained growth (Berüter and Droz, 1991; Archbold and Nosarszewski, 2004). Fluctuations in sorbitol and sucrose concentrations mirrored modeled changes in estimated whole-tree carbon balance, particularly in 2021 (**Figure 1**). Sorbitol, the major translocated sugar in apple, may therefore serve as a sensitive indicator of source–sink dynamics at the tree level (Nosarzewski and Archbold, 2007). In our data, significant reductions in sorbitol concentration coincided with abscission predictions according to the FSD model (**Figures 1**-**3**). Sorbitol does not accumulate to high concentrations in fruitlets. Sinks lack the capacity to synthesize sugar alcohols and imported sorbitol is rapidly metabolized due to high enzyme activity (Pavel and DeJong, 1995; Loescher and Everard, 2000; Li et al., 2012). This enzymatic conversion, or high metabolic strength, has been shown to be directly related to sink activity, i.e. the rate at which a fruitlet metabolizes assimilates through processes of converting sorbitol to hexoses or starch (Loescher and Everard, 2000; Yamaki, 2010; Tijero et al., 2021). Thus, fruitlets in the 90^th^ percentile accumulate higher concentrations of soluble sugars due to higher growth demand (**Figures 1**-**4**), resulting in increased turgor pressure in vacuoles and thus driving cell expansion further (Loescher and Everard, 2017).

Together with sink size, sink activity determines sink strength, a measure of the competitive ability of fruitlets to draw assimilates relative to other fruitlets or sinks in the tree (Yamaki, 2010). Our data revealed contrasting patterns of starch accumulation among fruit that highlighted differences in their sink strength. 10^th^ and 50^th^ percentile fruitlets showed little capacity to accumulate starch, whereas 90^th^ percentile fruitlets in the thinned treatment exhibited exponential starch accumulation, ultimately surpassing all other size classes (p < 0.001) (**Figure 1I**). In contrast, starch accumulation in the unthinned control did not diverge significantly between the 50^th^ and 90^th^ percentiles (**Figure 1J**). This was consistent with observed similarities in sugar concentrations and growth rates within the unthinned control (**Figures 1**, **4**). In fact, a large proportion of fruitlets in the 50^th^ percentile was predicted to set, although a higher proportion of this size class persisted through harvest (**Figure 5**). This discrepancy between FSD model predictions and observed persistence is likely due to fruitlet size variation within the 50^th^ percentile, which inherently captures greater variability in fruit weight. The FSD model required 2 more days to predict fruit set in the unthinned control, which aligns with the less pronounced size differences, sugar concentrations, and growth rates between 90^th^ and 50^th^ percentile fruitlets in the unthinned control. Overall, population differences between the 90^th^ and 50^th^ percentile were more pronounced in the thinned treatment and the FSD model accurately predicted the proportion of persisting fruitlets in each size class (**Figure 5**). Thus, thinner-induced carbon stress appeared to disproportionately impair the sink strength of 10^th^ percentile fruitlets and weaker fruitlets in the 50^th^ percentile, while 90^th^ percentile fruitlets were less affected or recovered more quickly (Goffinet et al., 1996), leading to an earlier thinning response and predictability by the FSD model. These observations support the generally accepted construct that chemical thinning is selective, favoring larger fruitlets.

In tissues of rapidly expanding young organs such as developing fruit, sucrose is preferentially hydrolyzed to glucose and fructose by invertase. This reaction effectively doubles the number of osmotically active molecules, driving turgor-dependent cell expansion and providing substrates for respiration and biosynthesis (Loescher and Everard, 2017). Consistently, fructose and glucose were accumulated at higher rates in fruitlets of 90^th^ percentiles supporting that fructose is a major contributor to growth processes because of its strong osmotic role and sustained accumulation during development (Berüter, 2004; Tijero et al., 2021; Li et al., 2018). Thus, sink activity encompasses both metabolic sinks, which utilize sugars through enzymatic conversion and storage, and osmotic sinks, which accumulate sugars in vacuoles to sustain water uptake and cell expansion. The regulation of sucrose hydrolysis thereby regulates sugar accumulation patterns, sink strength, and developmental dynamics (Loescher and Everard, 2017; Li et al., 2018). In our study, spikes in relative growth rates often coincided with surges in sucrose concentration across years and cultivars (**Figures 1**–**4**). This response was more pronounced in sucrose compared to sorbitol and may reflect sucrose’s role as an upstream signaling molecule: sucrose surges can modulate hormonal networks, triggering growth as shown in rose (Rosa hybrida) (Barbier et al., 2015) and increase the osmotic potential of developing fruitlets.

Soluble sugar concentrations and starch content were generally lower in 2022 than in 2021, likely due to successive applications of thinners during the 2022 sampling period (**Figures 2**, **3**). The decision to apply a second thinner seven days after the first was guided by the FSD model, which predicted a limited thinning response from the initial application (Hillmann et al., 2025). This was supported by the MaluSim model, which predicted a whole-tree carbon surplus the day of the first application, a condition that favors fruitlet retention due to reduced thinner efficacy (Lakso et al., 2006; Robinson et al., 2010; Lordan et al., 2020). Existing carbon balance models (Lakso et al., 2006; Pallas et al., 2016) that simulate the source–sink balance of individual apple trees have been extended to predict thinner efficacy thereby suggesting specified orchard management approaches regarding chemical thinner rates. At the fruitlet level, prior studies focusing on carbohydrate availability and fruitlet retention report variable outcomes depending on fruitlet size, position, and developmental stage (Marini, 2003; Ackerman and Samach, 2015; Jakopic et al., 2016). In our study, both cultivars showed synchronized reductions in sugar concentrations and growth rates of 10^th^ percentile fruitlets around 10 DAT in 2022, coinciding with peaks in abscission despite positive predicted carbon balance (**Figures 2**–**5**).

The use of plant growth regulators such as NAA to thin fruit further illustrates the role of carbohydrate stress in fruit abscission. Early studies demonstrated that the thinning effect of NAA is linked to reduced availability of reducing sugars (such as glucose and fructose) in developing fruit (Schneider and Lasheen, 1973; Stopar et al., 1997; Yuan and Greene, 2000; Zhu et al., 2011; Pleyerová et al., 2022). In the present study, a reduction in sorbitol concentration occurred following applications of NAA, but not in fruitlets of the unthinned control (**Figures 1G, H**). Increased dark respiration after application of NAA plausibly elevate maintenance and stress-related metabolic costs rather than enhanced biosynthesis (Untiedt and Blanke, 2001) in addition to slight photosynthesis depression following NAA application (Stopar et al., 1997; Zhu et al., 2011) leading to potential carbohydrate deficits. In 2021, 10^th^ percentile fruitlets consistently had lower concentrations of soluble sugars and starch and never recovered from the marked reduction in sorbitol concentration that ultimately led to an abscission prediction at 6 DAT (**Figures 1I**, **4B**). Fruitlets in the 10^th^ percentile have limited capacity to buffer carbon stress and likely slow growth to critically low levels leading to abscission, reflecting their constrained enzymatic ability to sustain metabolism under competitive conditions. Consistent with this, fruitlets deprived of photosynthate exhibit diminished capacity to resume import, indicating that carbohydrate-utilizing enzyme activity is compromised (Minchin et al., 1997).

Soluble sugar concentrations in the 90^th^ and 10^th^ percentiles generally reflected the proportion of fruitlets predicted to persist or abscise. Carbohydrate levels in the 50^th^ percentile were more variable, sometimes resembling the 90^th^ percentile and at other times aligning more closely with the 10^th^ percentile. This shifting pattern suggests that 50^th^ percentile fruitlets occupy a transitional state, with carbohydrate status fluctuating between that of strong sinks destined to persist and weaker sinks more likely to abscise. Thus, the simple one-step model of estimating fruitlet retention/abscission at ~50% growth of 90^th^ percentile fruitlets (Greene et al., 2013) is probably an appropriate approximation that explains whole-tree fruit set dynamics. The declines in soluble sugar and starch concentrations of ‘Gala’, especially of the 50^th^ percentile fruitlets, coincided with the FSD model’s forecast that 51% of fruitlets within the 50th percentile would abscise (**Figure 5**). This reinforces the link between reduced carbohydrate availability and heightened abscission risk in this intermediate group. Conversely in ‘Honeycrisp’, soluble sugar and starch content of 50^th^ percentile fruitlets aligned more closely with 90^th^ percentile fruitlets, an observation reflected in a higher proportion of setting fruitlets within the 50^th^ percentile (**Figure 5**). However, between the prediction date and harvest, more abscission occurred than was predicted by the model, and final fruit set was ~ 9% lower than predicted (Hillmann et al., 2025). Possibly, lower abscission rates in ‘Honeycrisp’ at the time of prediction (50%) compared to almost 90% in ‘Gala’, led to higher competition between fruitlets and more abscission later in the season. This suggests that the successive thinner applications induced nutritional stress that disproportionately slowed the growth of 10^th^ percentile fruitlets, as similarly observed in 2021 ‘Gala’, underscoring that fruit size and sink strength ultimately determine fruit survival (Marini, 2003; Botton et al., 2011). Further, early fruit drop during ripening just prior to harvest is an attribute of ‘Honeycrisp’ that potentially contributed to the disparity between the number of fruit after ‘June’ drop and harvest.

In addition to analyzing differences among different size classes, positional effects on sugar accumulation were assessed by comparing king and lateral fruitlets of a cyme in 2022. King fruitlets are typically larger and more likely to persist compared to laterals which are generally regarded as weaker sinks, especially in basal positions (Ferree et al., 2001; Botton et al., 2011; Jakopic et al., 2015). Indeed, lateral fruitlets comprised most abscising fruitlets (**Figure 5**). Nonetheless, when laterals survive competition, their final size has been shown to approximate that of kings at harvest, with overall cluster load exerting greater influence on fruit size than positional identity (Serra et al., 2022; Meheriuk and Fisher, 1973). Our results reveal nuanced positional effects on fruit growth and sugar accumulation. Relative growth rates alone showed no consistent main effect of position. However, a significant interaction with fruit size (p = 0.021) indicated that positional differences were more pronounced in 90^th^ percentile fruitlets, reflecting the competitive advantage of king flowers within an inflorescence (**Figures 4**, **5**) (Losada and Herrero, 2013). In fact, king fruitlets, within the 50^th^ percentile more consistently persisted throughout the season than laterals in the same size class in both cultivars (data not shown). In ‘Honeycrisp’ king fruitlets in the 90^th^ percentile exhibited substantially higher growth rates than their lateral counterparts, whereas differences were negligeable in the 50^th^ percentile (**Figures 4E, F**). Sugar concentrations also differed significantly by position (p < 0.001), with king fruitlets generally maintaining higher concentrations than laterals in both cultivars (**Figures 2**, **3**). However, these differences depended upon fruitlet size: positional effects were only evident in the 10^th^ and 50^th^ percentile classes prior to thinner application, supporting our interpretation of the well-established concept that thinners disproportionately affect weaker fruitlets, consistent with observed growth rate patterns.

Growth rates of the 90^th^ percentile fruitlets remained high until 21 DAFB, when the FSD model predicted final fruit set, despite declining soluble sugars between 15 and 21 DAFB (**Figures 2**–**4**). Notably, in ‘Honeycrisp’ fructose concentrations in 10th percentile fruitlets did not diverge significantly from 90^th^ or 50^th^ percentile fruitlets until after the second application (**Figures 3C, D**). This lack of early separation in sugar status is consistent with the FSD and MaluSim model predictions, which indicated limited thinning response to the first application (Hillmann et al., 2025). In other words, the carbohydrate dynamics observed here underscore the models’ thinning forecasts and help to explain the reduced efficacy of the initial thinner. Interestingly, differences in sorbitol concentrations between size classes were more pronounced in lateral fruitlets, compared to king fruitlets, highlighting the role of sink strength between positions and size classes in assimilating photosynthates. Together, these patterns suggest limited enzyme activity and sugar conversion in weaker fruitlets, making them more directly constrained by the rate of sugar accumulation and metabolic utilization (Loescher and Everard, 2017). Moreover, growth of 10^th^ and 50^th^ percentile fruitlets responded more immediately to declining sugar concentrations, while 90^th^ percentile fruitlets consistently exhibited higher growth rates, thus stronger sink strength, greater assimilate draw, and higher storage capacity (Jakopic et al., 2015; Bangerth, 2000; Dal Cin et al., 2009; Ferree et al., 2001).

## Conclusion

Our results demonstrate a close alignment of soluble sugar concentrations and starch content with early fruitlet growth and abscission of apple. Across cultivars and years, small fruitlets in the 10^th^ percentile and weaker fruitlets in the 50^th^ percentile exhibited lower sugar concentrations and reduced growth rates prior to abscission. The FSD model accurately predicted abscission rates of apple, at times when soluble sugar concentrations reached critically low levels. The response of weaker sinks to a reduction of assimilates by chemical thinner applications was more immediate compared to stronger sinks. Fruitlets of the 90^th^ percentile maintained higher sugar concentrations and growth rates with a higher likelihood of retention. Positional differences were evident only when size effects were considered, supporting the view that fruit set is affected less by position alone and more by a complex interaction between size, sink strength and resource availability.

## Data Availability

The raw data supporting the conclusions of this article will be made available by the authors, without undue reservation.
